# Analysis of natural structures and chemical mapping data reveals local stability compensation in RNA

**DOI:** 10.1093/nar/gkaf565

**Published:** 2025-06-26

**Authors:** Robert L Cornwell-Arquitt, Riley Nigh, Michael T Hathaway, Joseph D Yesselman, David A Hendrix

**Affiliations:** Department of Biochemistry and Biophysics, Oregon State University, Corvallis, OR 97333, United States; Department of Biochemistry, University of Nebraska–Lincoln, Lincoln, NE 68503, United States; Department of Biochemistry and Biophysics, Oregon State University, Corvallis, OR 97333, United States; Department of Electrical Engineering and Computer Science, Oregon State University, Corvallis, OR 97333, United States; Department of Biochemistry, University of Nebraska–Lincoln, Lincoln, NE 68503, United States; Department of Biochemistry and Biophysics, Oregon State University, Corvallis, OR 97333, United States; Department of Electrical Engineering and Computer Science, Oregon State University, Corvallis, OR 97333, United States

## Abstract

RNA molecules adopt complex structures that perform essential biological functions across all forms of life, making them promising candidates for therapeutic applications. However, our ability to design new RNA structures remains limited by an incomplete understanding of their folding principles. While global metrics such as the minimum free energy are widely used, they are at odds with naturally occurring structures and incompatible with established design rules. Here, we introduce local stability compensation (LSC), a principle that RNA folding is governed by the local balance between destabilizing loops and their stabilizing adjacent stems, challenging the focus on global energetic optimization. Analysis of over 100 000 RNA structures revealed that LSC signatures are particularly pronounced in bulges and their adjacent stems, with distinct patterns across different RNA families that align with their biological functions. To validate LSC experimentally, we systematically analyzed thousands of RNA variants using DMS chemical mapping. Our results demonstrate that stem folding, as measured by reactivity, correlates with LSC (*R*² = 0.458 for hairpin loops) and that instabilities show no significant effect on folding for distal stems. These findings demonstrate that LSC can be a guiding principle for understanding RNA function and for the rational design of custom RNAs.

## Graphical Abstract

**Graphical Abstract:**
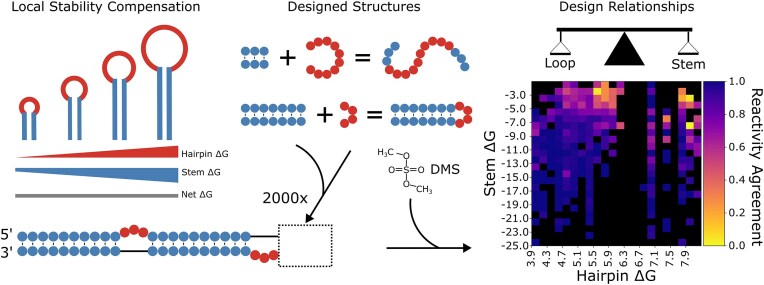

## Introduction

RNA molecules adopt complex structures that perform essential functions across biology, from regulating gene expression to modifying chromatin structure [1–3]. This structural versatility has inspired bioengineers to harness RNA as a platform for creating synthetic molecular machines, including scaffolds that colocalize enzymes [4–6], custom-designed ribozymes [7–9], and aptamers with specific ligand-binding properties [10–12]. The ability of RNA to perform these varied functions fundamentally depends on its ability to fold into three-dimensional structures. Therefore, advancing our understanding of RNA folding mechanisms is crucial for elucidating the behavior of biological RNAs and developing more effective RNA-based therapeutics and engineering applications.

The field utilizes diverse experimental approaches to understand RNA folding, ranging from high-resolution techniques like X-ray crystallography [13–15] and cryo-electron microscopy [16–18] to lower-resolution methods like chemical mapping [19–21] and cross-linking [22–24]. Yet fundamental challenges remain in the prediction of an RNA’s base-pairing pattern (secondary structure) from sequence alone, which would reduce the need for time-consuming experimental characterization of each new RNA [25]. Significant progress in computational prediction began with the development of the “Turner parameters,” which formed the foundation for algorithms like Mfold [26], RNAStructure [27], and RNAfold [28] that calculate the global minimum free energy (MFE) structure. The field continues to evolve rapidly, with linear-time structure prediction methods [29], machine learning [30], and deep learning approaches enhancing our ability to predict RNA secondary structures with increasing accuracy [31–33], but they often struggle when presented with novel RNAs [34] or RNAs from families that are different from what they were trained on. Therefore, the identification of new patterns that could improve RNA structure prediction remains highly valuable.

Structure prediction algorithms can also be leveraged to identify sequences that will fold into a desired secondary structure for engineered applications. This “inverse-folding” problem is computationally intensive due to the vast sequence space available for any base-pairing arrangement. Current approaches often focus on finding sequences that optimize the MFE [28, 35, 36]. However, these strategies result in sequences with high GC content, which often misfold [37]. The limitations of high-GC designs have been demonstrated empirically through the collective wisdom of Eterna participants, who found that excessive GC content diminishes the success of RNA design tasks across thousands of experiments [38]. Notably, nature does not employ this strategy—ribosomal and transfer RNAs (tRNAs) maintain consistent GC content despite variation in background GC content [39]. This observation suggests that natural RNA sequences follow alternative optimization principles that could inform more effective approaches to designing synthetic RNA structures.

The MFE approach considers the global sum of energy terms and fails to account for the spatial distribution of stability within an RNA structure. For example, larger RNA loops have been hypothesized to require larger stabilizing helices to offset the entropic cost of loop formation [40]. By simply minimizing free energy globally, this energetic requirement may be satisfied by helices located anywhere in the structure. Nature may allocate its limited free energy “budget” according to functional priorities and to specific locations in need of greater stabilization rather than global minimization. We propose that the local energetic balance between loops and their adjacent stems—a relationship we term local stability compensation (LSC)—may be a design rule that nature employs and could be leveraged in rational RNA design.

We propose that larger and more destabilizing loops in structures exhibiting LSC must be paired with proportionally larger and more stabilizing stems to ensure consistent folding for functional RNAs that rely on robust structures. However, LSC may not be strictly necessary for RNAs that lack evolutionary pressure for robust folding or for dynamic RNA substructures that adopt many conformations. Therefore, apart from such examples, we expect to observe a pattern where decreasing stem Δ*G* balances increasing loop Δ*G* to maintain a consistently negative net free energy (Δ*G*) (Supplementary Fig. S1A). Crucially, this compensation operates locally: each loop is primarily stabilized by its adjacent stems, with minimal influence on distant structural elements.

In this study, we first analyzed LSC signatures in natural RNA structures, revealing distinctive patterns in stem and loop Δ*G* distribution across different loop types and RNA classes. We then experimentally tested the relationship between LSC and its impact on folding by performing DMS (dimethyl sulfate) chemical mapping on a library of designed RNAs. Our results demonstrate that folding fidelity correlates with LSC and confirm that structural instabilities primarily affect local folding. These findings have significant implications for RNA design strategies, suggesting that meeting local stability requirements is crucial for successful substructure folding. Furthermore, understanding the stability requirements of individual substructures could enhance RNA design by facilitating their assembly into larger, more complex structures.

## Materials and methods

### Analysis of net Δ*G* for local substructures with bpRNA-1m

For our large-scale analysis of RNA structures, we used the meta-database bpRNA-1m [41]. A smaller subset without structures of 90% or greater sequence identity, called bpRNA-1m90, was also used to avoid oversampling particular RNAs. Importantly, structural data in the database are accessible as structure type (.st) files that contain a breakdown of the subcomponents of the RNA structure to facilitate our analysis. We calculated the free energies of the individual substructures using the Turner 2004 parameters for RNA folding [42]. We define net Δ*G* as the sum of the stabilizing stem free energy and the destabilizing adjacent loop free energy. In the case of hairpin loops, there is only one stem/helix immediately adjacent to the loop. For two-way junctions (i.e. bulges and internal loops), we average the free energies of both stems on each side of the junction.

**Figure 1.**
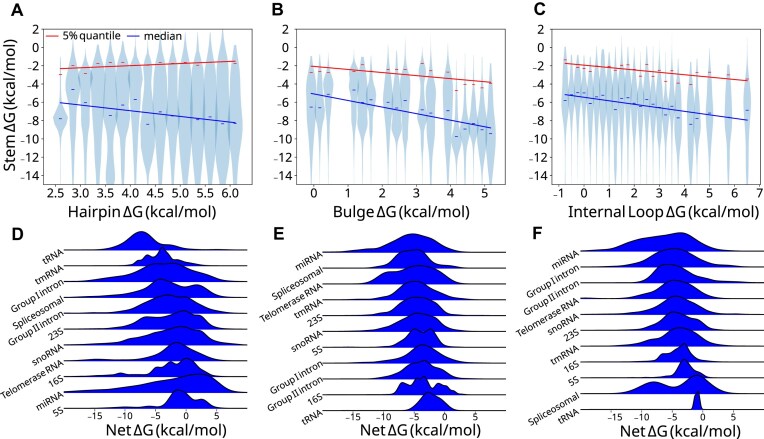
Local stability compensation in bpRNA-1m90. (**A**) Hairpin, (**B**) bulge, and (**C**) internal loop Δ*G*s from refolded bpRNA-1m90 structures are binned, and the corresponding stem Δ*G*s are plotted as violins with linear regressions drawn for the median (blue) and 5% quantile (red). bpRNA-1m90 net free energy kernel density estimates for (**D**) hairpin loops, (**E**) bulges, and (**F**) internal loops belonging to different RNA types.

We developed a pipeline using Vienna RNA to refold structures from the RNA families database and the comparative RNA web database using the consensus base pairs as hard constraints in order to correct for “underfolded” structures that contain large stretches of unpaired nucleotides. The resulting dataset was used for the analysis of large-scale trends across many RNAs (Fig. 1), and for correcting unrealistic loops in particular RNAs of interest (Fig. 3B–D). The dataset was not used when the numbering scheme of conserved loops needed to be preserved.

### Data analysis and statistics

All statistics were performed using Python code (https://doi.org/10.5281/zenodo.14252189) with the SciPy and Scikit-learn modules. For assessing the significance of net Δ*G* distributions in hairpin loops, bulges, and internal loops (Supplementary Fig. 1B), an in-structure rotation control was used: loop energies were summed with stem energies belonging to a distal substructure instead of loop-adjacent stem energies. This was only done for structures where more than three loops of a type existed to eliminate the possibility of a loop receiving one or more of its own stems. The difference over control in net free energy frequencies by bin was calculated along with an *f*-test to reveal the significance of differences in variance.

Three hundred ninety-two hairpin sequences were filtered out from the DMS reactivity results because of C repeats greater than four residues, which may result in unreliable DMS reactivities as seen in A repeats [43]. DMS data for 875 hairpin loops, 1979 bulges, and 1987 internal loops were available for analysis.

For analysis of DMS reactivity data, we employed the area under the receiver-operator curve (AUROC) to quantify folding fidelity, i.e. the agreement of reactivity values with the “designed” structure. This measure quantifies the overlap between the reactivity distributions of paired-by-design and unpaired-by-design nucleotides for a single RNA structure. In this framework, unpaired nucleotides are treated as the “positive predictions” and paired nucleotides are treated as the “negative predictions” for the AUROC calculation, while reactivity data provide the score to be compared. We calculated the AUROC using the Scikit-learn Python package for the AUROC score across adenine/cytosine (A/C) nucleotides in global, local, and distal scopes (all residues, residues in the residues region, and residues not in the randomized region, respectively). In addition, we measured the average reactivity of stems and loops belonging to the randomized region and a region distal to the randomized region as a control. Only reactivities corresponding to A or C nucleotides were used for these calculations. The sample sizes depended on the size of the substructure and the abundance of A/C residues (see library design for more details). Lastly, a set of per-position averages was calculated with 95% confidence for various bins of net Δ*G*, which involved sample sizes no lower than 70 nucleotides for each bin.

### Substructure free energy calculation and designed RNA library generation

We developed a Python module to calculate the free energies of folding by components called bpRNAStructure. We used bpRNAStructure to extract tab-delimited files containing bpRNA-1m data organized by RNA ID number and the substructure identifier. These data were used in the aforementioned analyses and statistics regarding bpRNA-1m. We also used bpRNAStructure to add the free energy component-wise as an additional annotation of the structure type file from bpRNA-1m, hence calling it a “ste” file for “structure type energy.” These files were used to organize data for each sequence in the designed RNA libraries, which are introduced below.

We used a Python script to randomly generate sequence libraries with an important set of constraints. Each sequence was based on a core template and required a Hamming distance of at least 20 compared to any other sequence, loops of A and C residues, and substructure lengths of 4–12, 3–11, 1–9, and 1–9 residues for stems, hairpin loops, bulges, and internal loops, respectively. The template was a triply bulged hairpin structure, with variable and semi-constant regions depending on the loop type being studied (Fig. 4A). The variable regions consisted of loops varying by length and stems varying by length, GC content, and G:U pairs. “Constant” energy stems were used for nonvarying (distal) regions and were designed to minimize free energy variation while allowing for sequence variation. Constant energy stems used G:C or C:G closing base pairs, a fixed GC content, and zero G:U pairs. Because sequences were required to be no shorter or longer than 20% of the mean sequence length, 21 and 13 sequences were filtered out of the internal loop and bulge libraries, respectively. Oligonucleotide pools of each library were ordered from Agilent Technologies.

### PCR amplification of DNA templates

DNA templates for transcription were prepared by polymerase chain reaction (PCR) amplification of the synthesized oligonucleotide pool. We first resuspended the oligo pool in 50 μl of IDTE buffer (1×, pH 8.0, IDT, #11-05-01-13). PCR amplification was performed using custom primers obtained from IDT: a forward primer containing the T7 promoter sequence (TTCTAATACGACTCACTATAGG) and a reverse primer (GTTGTTGTTGTTGTTTCTTT). Each 50 μl PCR reaction mixture contained Q5 High-Fidelity DNA Polymerase (25 μl, NEB, #M0494S), oligo pool template (2 μl), forward and reverse primers (2.5 μl each at 10 μM, diluted from 100 μM stocks), and RNase-free UltraPure water (18 μl, Thermo Fisher, #10977015). The PCR thermal cycling conditions consisted of initial denaturation at 98°C for 30 s, followed by 20 cycles of denaturation at 98°C for 10 s, annealing at 62°C for 15 s, and extension at 72°C for 15 s. A final extension step was performed at 72°C for 5 min. The PCR products were resolved by electrophoresis on a 2% agarose gel (150 V for 1 h) and extracted using the Zymoclean Gel DNA Recovery Kit (Genesee Scientific, #11-301C).

### *In vitro* transcription of RNA libraries

For *in vitro* transcription of the RNA libraries, we first prepared 10× Transcription (Tx) Buffer containing 400 mM Tris base, 10 mM spermidine, and 0.1% Triton X, 25 mM NTPs, 50 mM dithiothreitol (DTT), and 250 mM MgCl_2_. The transcription reaction contained 10 μl of 10× Tx Buffer, 5 μl of 50 mM DTT, 16 μl of 25 mM NTP solution, 8 μl of 250 mM MgCl_2_, 4 μl of T7 RNA polymerase (New England Biolabs, #M0251S), and 33 μl RNase-free water. Purified template DNA was quantified using a NanoDrop spectrophotometer, adjusted to 0.3 μM, and 24 μl was added to complete the reaction mixture. The complete reaction was incubated at 37°C for 6 h. Following transcription, the template DNA was removed by DNase I digestion using the RNA Clean & Concentrator-5 with DNase I kit (Genesee Scientific, #R1014). The RNA product was purified using the same kit according to the manufacturer’s instructions. Prior to DMS MaPseq analysis, we performed quality control by measuring RNA concentration by a NanoDrop spectrophotometer and confirming the RNA length by electrophoresis on a 4% denaturing agarose gel run at 150 V for 1 h.

### DMS probing of RNA libraries

For each RNA library, we prepared a solution containing 10 pmol of RNA in 5 μl RNase-free water. RNA samples were denatured by heating to 90°C for 4 min, followed by rapid cooling to 4°C for 3 min in a thermocycler. The denatured RNA was then added to a folding buffer containing 16.5 μl of 0.4 M sodium cacodylate buffer and 1 μl of 250 mM MgCl_2_, yielding final concentrations of 264 mM sodium cacodylate and 10 mM MgCl_2_ during the DMS reaction. The RNAs were folded at room temperature for 30 min. A fresh DMS solution was prepared by combining 15 μl DMS (Sigma–Aldrich, #D186309) with 85 μl 100% ethanol (Decon Labs, #2716). Immediately following the 30-min folding period, 2.5 μl of the freshly prepared DMS solution was added to the RNA–buffer mixture to initiate modification. The reaction was allowed to proceed for 6 min before being quenched with 25 μl BME (Thermo Fisher, #125470010). Modified RNA was purified using the RNA Clean & Concentrator-5 Kit (Genesee Scientific, #R1014) and eluted in 7 μl of RNase-free water. Final RNA concentration was determined using the Qubit RNA BR Assay Kit (Thermo Fisher, #Q10211), using 1 μl of the purified RNA sample for measurement.

To detect adenine and cytosine methylation sites, we employed Marathon reverse transcriptase (Kerafast, #EYU007), which incorporates mutations during complementary DNA (cDNA) synthesis at methylated positions. Prior to reverse transcription, we prepared the following solutions: 5× Marathon buffer (250 mM Tris–HCl, pH 8.3, 375 mM KCl, 15 mM MgCl_2_), 10 mM dNTPs, and 100 mM DTT. The modified RNA sample was diluted to 0.25 μM, and the RT primer (Supplementary Document: Sequences.xlsx) was diluted to 0.285 μM. The reverse transcription reaction was assembled by combining 3 μl of 5× TGIRT buffer, 1.49 μl of 10 mM dNTPs, 0.74 μl of 100 mM DTT, 1.2 μl of Marathon reverse transcriptase, 6.4 μl of diluted modified RNA, and 1 μl of diluted RTB primer. The reaction was thoroughly mixed and incubated at 42°C for 3 h. Following incubation, we added 5 μl of 0.4 M NaOH to the reaction and subjected the mixture to a denaturation step (90°C for 4 min, followed by cooling at 4°C for 3 min). The NaOH was then neutralized by adding 2.02 μl of Quench acid (1.43 M NaCl, 0.57 M HCl, 1.29 M sodium acetate). To purify the cDNA product, we first added 27.5 μl of RNase-free water to the neutralized reaction, and then purified using the Oligo Clean & Concentrator Kit (Genesee Scientific, #11-380B). The final cDNA product was eluted in 15 μl of RNase-free water.

The cDNA products were amplified by PCR using primers obtained from IDT (dissolved in IDTE, pH 8, buffer at 100 μM). We used the forward primer AATGATACGGCGACCACCGAGATCTACACTCTTTCCCTACACGACGCTCTTCCG and the reverse primer CAAGCAGAAGACGGCATACGAGATCGGTCTCGGCATTCCTGCTGAACCGCTCTTCCGATCTGGTACTATGTACCAAAG. Prior to PCR setup, primers were diluted 1:10 to 10 μM in RNase-free water. The PCR reaction contained 25 μl Q5 High-Fidelity DNA Polymerase (New England Biolabs, #M0494S), 5 μl each of diluted forward and reverse primers, 6 μl purified cDNA, and 9 μl RNase-free water. Thermal cycling conditions consisted of an initial denaturation at 98°C for 30 s, followed by 16 cycles of denaturation at 98°C for 10 s, annealing at 62°C for 15 s, and extension at 72°C for 15 s. A final extension step was performed at 72°C for 5 min. For gel purification, we first prepared a 2% agarose gel by diluting 10× Tris-borate-EDTA (TBE) buffer (Bio-Rad, #1610770) to 1× with RNase-free water, and then dissolving 1.5 g agarose (Apex, #20-102) in 75 ml of 1× TBE buffer. The solution was heated in a microwave for 2 min, mixed thoroughly, and supplemented with 10 μl SYBR Safe DNA Gel Stain (Thermo Fisher, #S33102) before casting. The Fisherbrand Horizontal Electrophoresis System (Fisherbrand, #FBSB710) was used for electrophoresis, with 1× TBE as a running buffer. PCR products were mixed with 10 μl TackIt Cyan/Yellow Loading Buffer (Thermo Fisher, #10482035) and separated on the gel at 150 V for 60 min. After imaging, bands of the correct size were excised and purified using the Zymoclean Gel DNA Recovery Kit (Genesee Scientific, #11-301C). Final DNA concentration was determined using the Qubit 1× dsDNA High Sensitivity Assay Kit (Thermo Fisher #Q33230).

### DMS-MaPseq data analysis

Sequencing was performed on a NovaSeq 6000. Each sequencing run was first demultiplexed using the RTB barcodes added during the RT. Demultiplexing was performed using novobarcode (https://www.novocraft.com/documentation/novobarcode/demultiplexing-barcodedindexed-reads-with-novobarcode/).

novobarcode -b rtb_barcodes.fa -f test_R1_001.fastq test_R2_001.fastqwhere rtb_barcodes.fa gives a list of sequencing barcodes, such as

Distance 4

Format 5

RTB021 CCAATGGGTGTA

RTB022 AGCCAAAACTGG

RTB023 GTGTGTTTGCCC

Where three barcodes are specified, distance is the distance in base pairs between a barcode and an allowable read. Format specifies that the barcode will be on the 5′ end of read 1.

Lastly, we used the RNA mutational profiling (RNA-MaP) tool to count mutations to determine the mutational fraction at each nucleotide position (https://github.com/YesselmanLab/rna_map). This is an open-source tool developed to simplify mutational profiling analysis.

rna-map -fa test.fasta -fq1 test_mate1.fastq -fq2 test_mate2.fastq --dot-bracket test.csv --param-preset barcoded-libraries

The rna-map command requires a FASTA-formatted file containing all DNA reference sequences and the paired sequencing reads generated from the previous step. We also supplied a CSV file containing the dot bracket structure for each RNA with the --dot-bracket flag and applied stricter constraints to how well each sequence needs to align to a read --param-preset barcoded-libraries. All the data are stored in JSON format in a pandas DataFrame.

## Results

### Evidence for local stability compensation found in a structural meta-database

LSC proposes that RNA folding stability requires larger, destabilizing loops to be paired with more stabilizing adjacent stems (Supplementary Fig. S1A). To test whether LSC is observed in biological RNAs, we analyzed the relationship between loop and stem free energies (Δ*G*) across 79 582 hairpins, 30 441 bulges, and 17 117 internal loops in the bpRNA-1m90 database. Importantly, RFAM structures can feature unrealistically large loops originating from nucleotide stretches that do not align with the consensus structure. We applied a pipeline (see the “Materials and methods” section) to refold these loops holding consensus base pairs fixed resulting in targeted corrections that introduced shifts in the bulk distributions, particularly a depletion of the highest net Δ*G* loops (Supplementary Fig. S2). In the resulting data, hairpin loop, bulge, and internal loop Δ*G* bins of 0.25 kcal/mol correlate negatively with the median stem Δ*G* (Fig. 1A–C), with bulges appearing to correlate more strongly (*R*^2^ = 0.647) (Supplementary Table S1). In order to assess the upper limit of stem free energy for associated loops, we compared the 5% weakest stem Δ*G*s (Fig. 1A–C). The 5% quantiles for bulges and internal loops correlate with a slope similar to the median, except for hairpins. Furthermore, bulge and internal loop free energy sum (net Δ*G*) distributions are enriched between −3 and −6 kcal/mol compared to a control in which stems are swapped with distal stems (see the “Materials and methods” section), which is not observed in hairpins (Supplementary Fig. S1B). These data suggest that LSC exists in bpRNA-1m90 structures, but is partially obscured in hairpins, likely by RNA family-specific factors that may contribute to the inconsistent compensation patterns. It is likely not all RNAs have strong structure requirements, and we may see stronger effects examining only RNAs in a given family.

When grouped by their respective RNA family, net Δ*G* comparisons of hairpins (Fig. 1D), bulges (Fig. 1E), and internal loops (Fig. 1F) revealed distinct stability compensation patterns. Hairpin loops showed the highest variability, with a median net Δ*G* of −2.66 kcal/mol and a standard deviation of 1.74 kcal/mol, which is higher than that of internal loops (1.33 kcal/mol) and bulges (0.75 kcal/mol) (Supplementary Table S2). A comparison by more specific RNA subclasses (Supplementary Fig. S3) corroborated this observation, albeit with greater variation (Supplementary Table S2). Because the variance by RNA type accounts for some portion of the variance unexplained by the global correlation, and the aforementioned variances by type are inversely proportional to the corresponding global correlations, RNA family-specific compensation patterns are in part responsible for deviations from LSC.

### Local stability compensation varies by RNA type and is consistent with function

**Figure 2.**
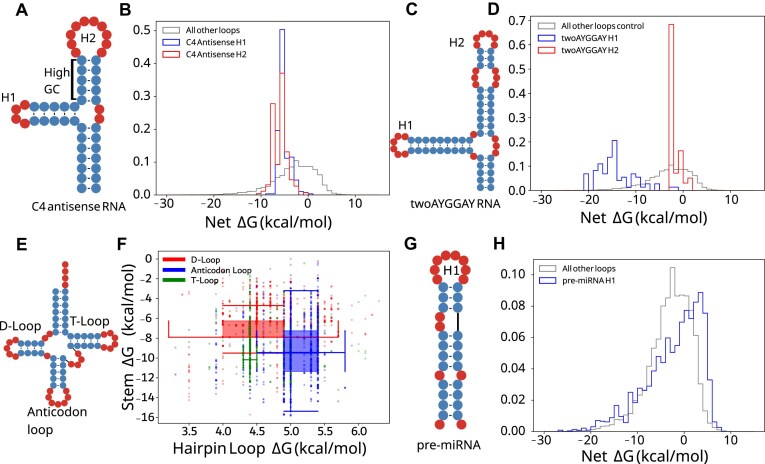
Net Δ*G* distributions are distinct for RNA types and particular RNA loops. (**A**) C4 antisense RNA example secondary structure and (**B**) hairpin net Δ*G* distribution (H1 in blue, H2 in red) and other hairpins in bpRNA-1m90 (gray). (**C**) TwoAYGGAY RNA example secondary structure and (**D**) net Δ*G* distribution (H1 in blue, H2 in red) and other hairpins in bpRNA-1m90 (gray). (**E**) Example tRNA secondary structure (bpRNA_RFAM_1424) and (**F**) two-dimensional box plots for the D-loop, T-loop, and anticodon loop in red, green, and blue, respectively. (**G**) pre-miRNA example secondary structure and (**H**) net Δ*G* distribution (blue) and other hairpins in bpRNA-1m90 (gray).

We hypothesized that LSC may be more prominent in specific RNA families where high stability is needed. Conversely, we speculated that high net Δ*G* for some families may indicate functions where destabilization is necessary. To investigate in more detail, we highlight four classes with distinct LSC behaviors. The C4 antisense RNA is a noncoding RNA (ncRNA) contained in P1 and P7 bacteriophages [44, 45] (Fig. 2A). Hairpin loops within the C4 antisense RNA were selected for further investigation because they have negative and highly conserved net Δ*G*s. The C4 antisense RNA contains two highly stabilized hairpins. H1 consists of a highly stable hairpin loop and stem, while H2 consists of a large hairpin loop and stable GC-rich stem. Both stem-loops achieve highly similar net Δ*G*s despite the substantial differences in their subcomponents (Fig. 2B).

The TwoAYGGAY RNA motifs (Fig. 2C), characterized by two conserved AYGGAY terminal hairpin loops [45], provided an example of family-specific stability patterns. In a departure from the expectations of LSC and the observations in C4 antisense RNAs, the TwoAYGGAY RNAs showed a bimodal net Δ*G* distribution, with peaks at −16 and −3 kcal/mol (Fig. 2D and Supplementary Fig. S3A), revealing that identical hairpin loop sequences can maintain different stabilities. This conserved differential stabilization within the same RNA structure suggests a potential functional significance for this relatively unexplored motif. This RNA class demonstrates that LSC patterns may be of interest in RNAs of unknown function.

tRNAs are a well-studied class of ncRNAs that are involved in translation with a cloverleaf secondary structure containing three hairpins (Fig. 2E). We investigated both the stem and loop free energies in tRNAs for energy relationships specific to different hairpins. tRNA hairpins group into three distinct regions of stem and hairpin loop Δ*G*, shown with box and whisker plots (Fig. 2F). While the D-loop and anticodon loop have similar variability, the anticodon loop is larger and higher energy and is also associated with a more stabilized stem. The observation of more destabilizing loops being coupled with more stabilizing stems is consistent with the LSC hypothesis.

MicroRNA (miRNA) precursors (pre-miRNAs) have well-defined structures (Fig. 2G) that are conserved for the biogenesis of small RNAs and share a similar function despite substantial sequence and structure variation [46, 47]. Indeed, pre-miRNA hairpins in bpRNA-1m90 show a wider net Δ*G* distribution than all other types (Fig. 2H), which may be consistent with the target sequence diversity of miRNAs [47] and the loop positioning important for Dicer function [48]. These structures frequently contained poorly stabilized hairpin loops with net Δ*G*s reaching 5 kcal/mol, far higher than the hairpins typical of other substructures (Fig. 2H). This high instability of the stem closing the hairpin loop is notable due to its proximity to the cleavage site. Because of phylogenetic differences in miRNA maturation, we continue this analysis of pre-miRNAs from a phylogenetic perspective.

### Local stability compensation is distinguished by phylogeny

**Figure 3.**
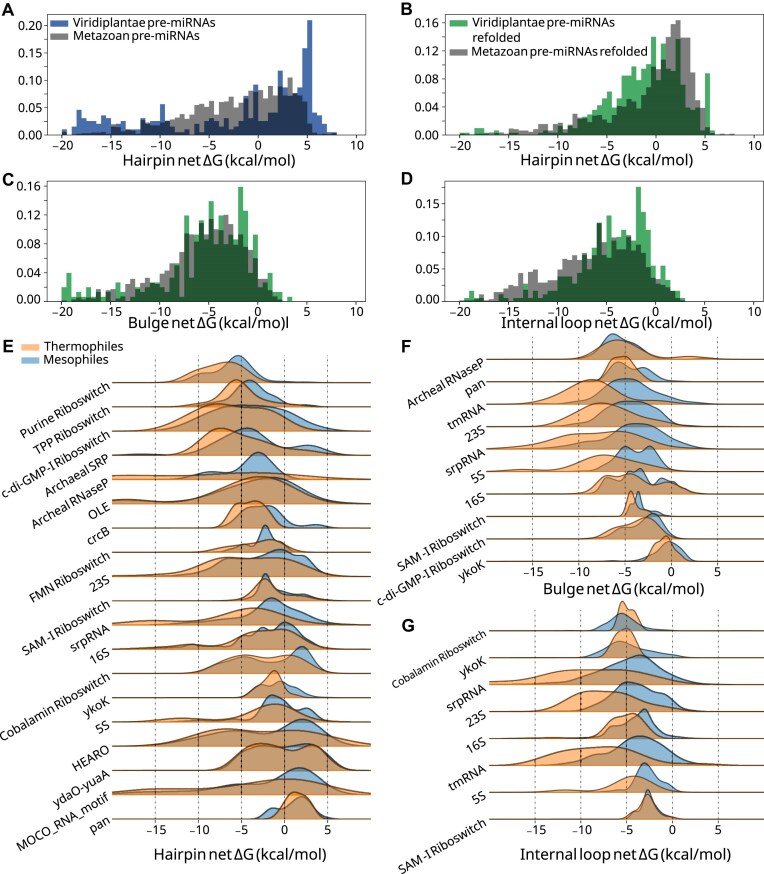
Differences in net Δ*G* by phylogeny for select RNA types. Plant pre-miRNA hairpin net Δ*G*s (green) and metazoan (gray) net Δ*G*s (**A**) before folding with constraints and (**B**) after folding with constraints. (**C**) Plant pre-miRNA bulge Δ*G*s (green) and metazoan (gray) net Δ*G*s after folding with constraints. (**D**) Plant pre-miRNA internal loop Δ*G*s (green) and metazoan (gray) net Δ*G*s after folding with constraints. (**E**) Thermophilic (orange) hairpin net Δ*G*s and non-thermophilic net Δ*G*s (blue). (**F**) Thermophilic (orange) bulge net Δ*G*s and non-thermophilic net Δ*G*s (blue). (**G**) Thermophilic (orange) internal loop net Δ*G*s and non-thermophilic net Δ*G*s (blue).

Owing to species-related differences in form and function of miRNAs, and to the thermodynamic nature of LSC, we investigated how LSC correlates with phylogeny. We compared plant and metazoan pre-miRNAs, as well as several RNA types belonging to thermophiles and non-thermophiles. Plant pre-miRNA hairpins were enriched relative to metazoan pre-miRNAs at 5.0 kcal/mol and above (Fig. 3A and Supplementary Table S3), but these structures included many hairpin loops of unrealistic sizes. To account for this, we employed the results of structure prediction with constraints (see the “Materials and methods” section). In the constrained folding data, plant pre-miRNA hairpin loops are significantly more stabilized than metazoans (Fig. 3B and Supplementary Table S3), suggesting a distinguishing role of apical hairpin loop stability in metazoan miRNAs. While bulges are not significantly distinguished (*P* = .05), plant pre-miRNA internal loops are less stabilized than those of metazoa (Fig. 3C and D, and Supplementary Table S3). These observations are consistent with the role of loop-distal regions [49] and duplex stability [50] in modulating plant pre-miRNA processing.

Thermophilic species exhibit greater hairpin, bulge, and internal loop stability in ncRNAs relative to their mesophilic counterparts, in alignment with previous research showing increased GC content in tRNAs and rRNAs [39] (Fig. 3E–G). There are exceptions for which differences in local stability are either minute or absent, including regulatory elements such as the YkoK leader, ydaO, and the cobalamin riboswitch. The functional roles of participating substructures may explain the similarity in net Δ*G* for these structures. Interestingly, the *S*-adenosylmethionine riboswitch (SAM-I) shows strong agreement between thermophilic and non-thermophilic species for hairpins and internal loops (Fig. 3E and G), yet a lower net Δ*G* exists for thermophilic SAM-I bulges (Fig. 3F). The only SAM-I bulge known to vary across phylogeny is a bulge adjacent to the SAM binding pocket, which is suggested to indirectly stabilize the pocket and promote the adoption of the binding-competent conformation [51]. Deletion of this bulge is highly deleterious in *Bacillus subtilis*, but not in *Thermoanaerobacter tengcongensis*, a thermophilic species, which may stabilize the binding pocket by other means such as a base triple [51]. Thus, in an otherwise highly conserved riboswitch, a substructure has a lower net Δ*G* in thermophiles than it does in mesophiles. In light of these observations and in order to bypass confounding biological context, we devised an experiment to test LSC in rationally designed constructs.

### DMS reactivity of three sequence libraries demonstrates local stability compensation

**Figure 4.**
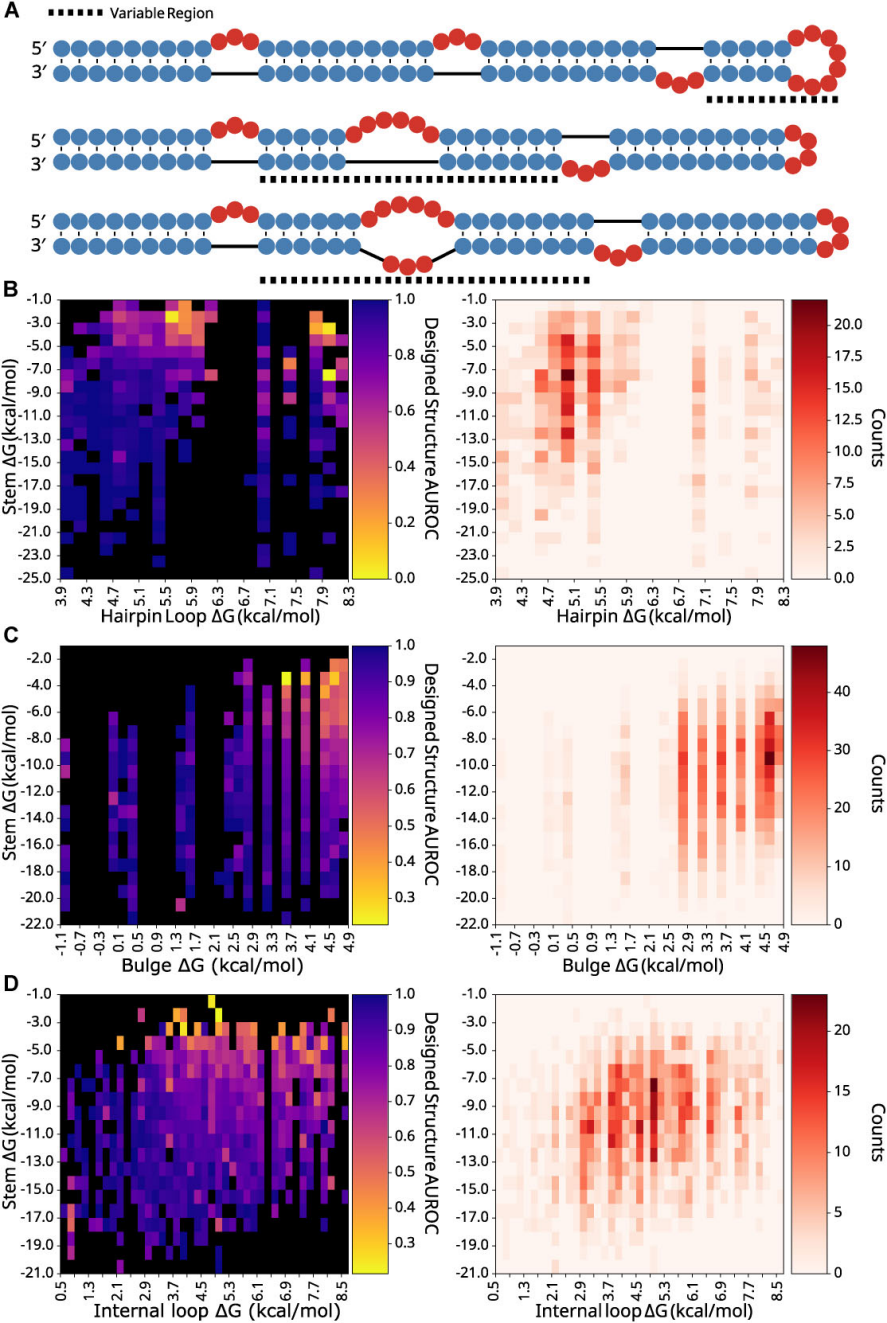
Library design and local structure AUROC heatmaps. (**A**) Designed structure examples for hairpins, bulges, internal loops (top, middle, and bottom, respectively); dashed lines indicate the variable region. Average AUROC and structure count heatmaps for cells of (**B**) hairpin, (**C**) bulge, and (**D**) internal loop Δ*G* by adjacent stem Δ*G*; yellow cells show low AUROC or poor agreement of DMS data with the designed structure, while dark blue cells show high AUROC with strong agreement, and black cells contain no data. Count heatmaps show the number of RNAs contributing to each cell with darker red coloring indicating a higher number of RNAs.

To directly test LSC, we designed three RNA libraries that systematically varied local stabilities for assessment with DMS chemical mapping. Each library used a template structure containing three bulges and one stem-loop, with randomized sequences in specific regions to generate variants with different local stabilities (Fig. 4A). This design allowed us to measure how stability changes affect both local and distal regions. To isolate the effects of local stability changes, we engineered constant-energy stems outside the variable regions as internal controls for our reactivity measurements.

Each library showed acceptable coverage of the distribution of net Δ*G*s in bpRNA-1m90 (Supplementary Fig. S4A, C, and E). It is noted that the highest net Δ*G* bulges are underrepresented in the library when compared to their natural frequency in bpRNA-1m90. We used RNAfold [28] to predict each structure in the libraries and used the structure alignment approach bpRNA-align [52] to calculate a structural similarity score between each designed structure and its corresponding predicted structure, revealing that RNAfold correctness for the designed structures is unimpacted by loop Δ*G* (Supplementary Fig. S4B, D, and F). The designed RNA structures were synthesized and DMS reactivity values were collected with a high-throughput sequencing approach (see the “Materials and methods” section).

To evaluate design success with a single metric, we used an established method to quantify the consistency between a given designed structure and the corresponding reactivity data [53]. This was achieved by calculating the AUROC of the DMS reactivity data against the designed target structure for each library (see the “Materials and methods” section). The values were binned by stem and loop free energy and are displayed in heatmaps (Fig. 4B–D). The heatmaps show lower average AUROC values for structures of higher loop Δ*G* and lower stem Δ*G* across each structure component type. In each heatmap, a roughly linear boundary exists past which local folding fidelity rapidly decreases, indicating that stem and loop energies both contribute to local folding fidelity. Furthermore, AUROC correlations are strongest with net Δ*G*, although stem Δ*G* correlates more than loop Δ*G* (Supplementary Table S4A). It is noted that a number of low stem energy, low loop energy bulges and internal loops have poor AUROC values. As a control for the nonlocal structure, the AUROC scores for the remaining region are unaffected by the net Δ*G* of the local, variable region (Supplementary Fig. S5).

### Loop stabilization primarily impacts adjacent stem reactivities

**Figure 5.**
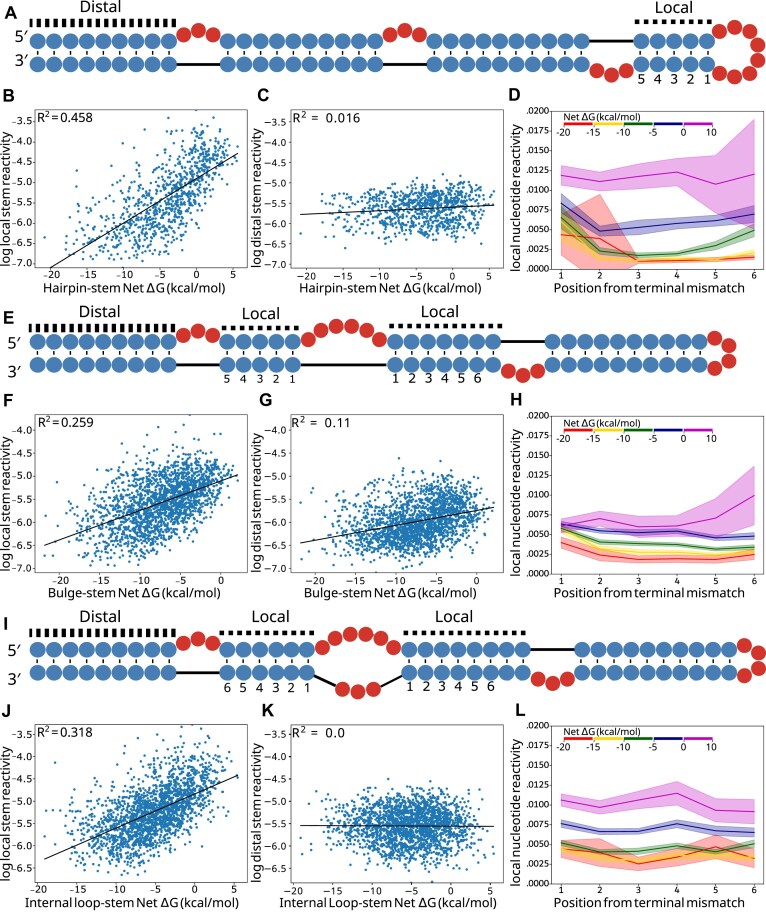
Local versus distal stem reactivity correlations. (**A**) Example structure from the hairpin library with adjacent and distal stems to the hairpin loop labeled and local stem base pair indices labeled. (**B**) Linear regression between hairpin net Δ*G* and log average local stem reactivity, compared to (**C**) average distal stem reactivity. (**D**) Average net Δ*G* bin reactivity per position away from the hairpin loop. (**E**) Example structure from the bulge library with adjacent and distal stems to the bulge labeled and local stem base pair indices labeled. (**F**) Linear regression between bulge net Δ*G* and log average local stem reactivity, compared to (**G**) average distal stem reactivity. (**H**) Average net Δ*G* bin reactivity per position away from the bulge, where an index is present on both stems; the average is used. (**I**) Example structure from the internal loop library with adjacent and distal stems to the internal loop labeled and local stem base pair indices labeled. (**J**) Linear regression between internal loop net Δ*G* and log average local stem reactivity, compared to (**K**) average distal stem reactivity. (**L**) Average net Δ*G* bin reactivity per position away from the internal loop, where an index is present on both stems; the average is used.

We investigated which components of the structures in the library were changing in low AUROC structures (Fig. 5). For both local and distal helices, we calculated the average stem reactivity, which is a more direct readout of helix formation than local AUROC. Locally, average stem reactivity correlates well with stem-loop net Δ*G* and shows a strong positive trend. Correlation *R*^2^ values were 0.458, 0.259, and 0.318 for hairpin loops, bulges and internal loops, respectively (Fig. 5B, F, and J). These correlations are stronger than loop or stem Δ*G*s alone (Supplementary Table S4B). This indicates that stems closing less stabilized loops either adopt partially folded states or completely unfold. Distal stem reactivity shows no correlation with net Δ*G* (Fig. 5C and K), except for a weaker but slightly positive trend in bulges (Fig. 5G). These data show that RNA stem-loop instabilities have predominantly local effects.

We next sought to investigate how insufficiently compensated stems were impacted at the residue level. We calculated the average DMS reactivity of each base pair succeeding the loop for stems belonging to five different net Δ*G* bins. Hairpins, bulges, and internal loop averages and 95% confidence intervals are plotted (Fig. 5D, H, and L) up to position 6, after which too few data points exist for the higher energy bins. For bulges and internal loops, values for positions present on both stems were averaged. In agreement with average stem reactivity results, hairpin loop, bulge, and internal loop per-nucleotide reactivity distributions increase monotonically with each Δ*G* bin. Stem reactivity increases more for the closing base pair (position 1) than for the subsequent pairs. Stems closing hairpin loops show this behavior more strongly and for lower free energy bins than those closing bulges and internal loops, possibly due to strain involved in loop closure for hairpins. Bins of higher net Δ*G* experience dramatic shifts in reactivity for the middle of the stem (positions 2–5), and for all loop types, the highest energy bin (ΔG > 0 kcal/mol) shows reactivity consistent with unpaired nucleotides for the entire stem. Importantly, the net Δ*G* range between −5 and 0 kcal/mol shows reactivity values that are intermediate between paired reactivities and unpaired reactivities. This intermediate category is the most represented in bpRNA-1m90 histograms, while fewer substructures in the database belong to the high-energy bin (0–10 kcal/mol), and still fewer belong to the lowest energy bin (−20 to −15 kcal/mol) (Supplementary Fig. S4A, C, and E).

To investigate other reactivity changes possibly responsible for the observed AUROC values, average loop reactivities were evaluated across stem-loop net Δ*G*s (Supplementary Fig. S6A–C), revealing weak trends toward lower reactivity with respect to stem-loop net Δ*G*. These trends are similar in strength and magnitude across loop types, and the trends for local loop reactivity are marginally stronger than those of the distal loop. It is expected that loop reactivities can significantly decrease when a bulge or internal loop shifts (Supplementary Fig. S7B). While these weak negative trends in loop reactivity do not compare to the correlations found between substructure net Δ*G* and stem reactivity, they partially contribute to local AUROC values. Thus, loop residues are not primarily responsible for changes in AUROC with increasing net Δ*G*;rather, it is the degree to which stems compensate for loop closure that produces the observed changes in local AUROC.

### Local substructure net free energies are correlated to local folding fidelity and fit the Hill equation

**Figure 6.**
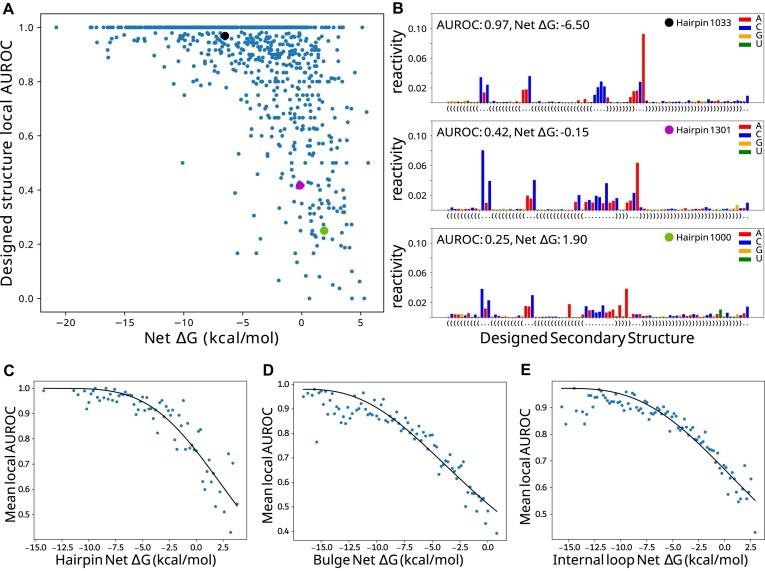
Local structure fidelity curve fitting. (**A**) Hairpin net Δ*G* versus average local AUROC; datapoints indicated by larger black, magenta, and cyan dots are shown in panel (**B**); raw reactivity values color-coded by nucleotide for three example designed hairpins; the boxed region is involved in AUROC calculation. (**C**–**E**) Hill equation curves fit to net Δ*G* bins (0.2 kcal/mol) versus the mean AUROC for each bin.

We next sought to define a relationship between LSC and local folding fidelity by comparing net Δ*G*s with the AUROC values for each structure type such that a given net Δ*G* estimates the resulting folding behavior. Highlighted in the hairpin loop scatterplot (Fig. 6A) are three examples showing high, medium, and low agreement with the designed structure (Fig. 6B). Structures with low AUROC (i.e. 0.25) contain many bases that were designed to be paired yet have higher reactivity than those of the adjacent loop.

In order to reduce noise associated with the DMS reactivity data and the AUROC calculation, AUROC data were averaged across bins of 0.2 kcal/mol net Δ*G*. The observed local AUROC asymptotically approaches 1.0 for decreasing net Δ*G*, suggesting a two-state model that includes a state that folds according to the design and a divergent folding state at low AUROC values. This observation of a two-state model with saturation led us to fit the averages to the Hill equation (Fig. 6C–E). Hairpin, bulge, and internal loop AUROC data cross an AUROC of 0.9, which represents minor DMS data disagreement, at −4, −9, and −6 kcal/mol, respectively (Fig. 6C–E). These thresholds show the least stability needed to fold with reasonable consistency. Hairpin AUROC data show a steeper curve (larger Hill coefficient) (Supplementary Table S5) and a clearer fit line, indicating that hairpin loops are less tolerant of reductions in stability compared to bulges and internal loops. Relative to hairpins and internal loops, bulges require a greater degree of stabilization, as indicated by the more negatively shifted curve. The consistency of the fit to the Hill equation suggests that net Δ*G* is, on average, an effective predictor of local folding fidelity.

## Discussion

Here we demonstrate that local stability compensation, wherein loops are stabilized by their adjacent helices, can be observed within an RNA meta-database and in DMS reactivity data collected for three structure libraries. The inherent diversity of structures and functions throughout RNA families leads to varying levels of LSC, and the clearest trends are observed for bulges and internal loops, while a wider range of net energies exists for hairpins. By studying three libraries of template-based RNA designs, we show a degree of independence of local stability on the remainder of the structure, and reveal how the net free energy of substructures influences the folding of the involved stem.

Differences in local substructure stability exist between RNAs of different form and function. Bulges, which showed the strongest correlation between stem and loop free energy, had the least variance in net free energy by RNA type, while the opposite was observed for hairpins. The structural and functional contexts of different RNA types also play a role in stability variation as the Turner parameters do not capture the free energy contributions of ligand binding, non-Watson–Crick–Franklin base pairs, and tertiary interactions—an important limitation of our study. Additionally, we averaged the free energies of flanking stems of bulges and internal loops, which is an efficient way to define a “local” substructure; however, this approach overlooks stem symmetry and obfuscates isolating one substructure from another. A more refined model for determining locality should be pursued to address this limitation.

Many RNA families show patterns that are consistent with LSC. The tRNA T, D, and anticodon loops were clustered into distinct stem and loop free energy regions (Fig. 2F). While the anticodon loop is typically larger, possibly to accommodate other interactions such as with the codon, it also shows a more stable associated stem, consistent with the LSC hypothesis. C4 antisense RNAs are characterized by two hairpin loops consistent with LSC: H2 is larger, and has an adjacent stem with high GC content, while H1 is a highly stable tetraloop and is closed by a stem with low GC content (Fig. 2B). Lastly, this observation may be applied to unknown structures. For example, the TwoAYGGAY motif has two identical hairpin loops that show distinct patterns of net free energy (Fig. 2D), suggesting different structural contexts that may be of interest for future research.

Such functionally relevant LSC patterns include taxonomy-specific distinctions. For example, metazoan microRNA hairpin loops exhibited an enrichment of the highest net Δ*G*s, which may be consistent with functions of the apical loop. DGCR8 [54] and hemin [55] interact at the UGU motif in the apical loop, as well as a peptide [56] designed to prevent miRNA maturation. Additionally, the instability of the stem adjacent to the hairpin loop may facilitate the adoption of the local helical distortion favorable for interactions with the dsRBD of DICER [57]. In contrast, plant pre-miRNAs, which do not involve the hairpin loop for maturation, do not exhibit high-energy hairpin loops, but instead show an enrichment of high-energy internal loops. Taken together, these observations suggest that substructure net free energy is linked to biological context. Furthermore, patterns in net free energy may provide clues to uncover functions and highlight biological differences that could drive future investigations.

The designed structure libraries revealed contributions of stem and loop free energy to folding fidelity, which solidifies net Δ*G* as an appropriate metric for LSC. The primary factor underlying a substructure’s folding fidelity was the reactivity of the involved stem or stems. Comparing these reactivities against those of distant stems revealed that LSC impacts the local stem much more than distal stem, suggesting that local substructures maintain a degree of independence or modularity on the thermodynamic level. Notably, the distal reactivities for bulges showed a detectable trend unlike the distal reactivities for hairpins and internal loops, which could be related to the underrepresentation of high-energy bulges (Supplementary Fig. S4). Loops were found to decrease in reactivity with respect to net Δ*G*, possibly due to the influence of noncanonical interactions or artifacts of successive reactive nucleotides [43].

The relationship between net Δ*G* and folding fidelity is half-sigmoidal, with substructure free energies around −20 to −10 kcal/mol plateauing at an AUROC of 1.0 (Fig. 6C–E). Such over-stabilized structures are very rare in naturally occurring structures, consistent with the idea of an “energy budget,” wherein G-C base pairs have a tendency to not be overused because of the diminishing returns as indicated by the plateauing trend. In contrast, intermediate net Δ*G* values (−10 to 0 kcal/mol) are abundant in naturally occurring structures. The reactivity data (Fig. 5D, H, and L) show intermediate reactivity across these substructures and may be explained by partial folding, transient folding, or likely an ensemble of folding states [58–60], which are implicated in RNA function. Under-stabilized structures with a net Δ*G* > 0 kcal/mol are infrequent and could possibly represent substructures that require instability or are stabilized by external interactions (e.g. microRNA hairpin loops interacting with hemin and DGCR8) or tertiary interactions. Tertiary motifs involve nonlocal interactions and were not included in the study; however, it is likely that the local stability of participating substructures may influence tertiary motifs or their formation. Investigating the relationship between local stability compensation and tertiary contact formation would be a logical next step for future research.

In MFE RNA structure prediction, randomized sequences can fold with a similar MFE compared to the original sequence [61], and MFE is not distinguished between known structured RNA and random sequences [62]. Additionally, since the number of possible helices increases exponentially with length [61], it is clear that additional constraints are needed to improve thermodynamic structure prediction. Our results suggest that local free energy balance in addition to global minimum free energy will improve thermodynamic approaches for structure prediction as well as the inverse folding of RNA.

Here, we describe a thermodynamic locality that occurs fundamentally in RNA structure and has widespread implications for the rational design of RNA, where both sequence and structure are engineered to achieve a desired outcome. According to the presented relationships between LSC and local folding behavior, an RNA engineered from well-folding substructures may also be expected to fold well, and an otherwise stable RNA may include intermediate to under-stabilized substructures, since the effects of this instability on the remainder of the structure are mostly insignificant (Fig. 5 and Supplementary Fig. S5). Further work with RNA libraries and high-throughput chemical mapping could reveal similar relationships in multiloops and other RNA motifs, as well as uncover the role of LSC in small binding sites. With further characterization, LSC could serve as a guide for the design of future biotechnology, including molecular machines composed of structured RNAs.

## Supplementary Material

gkaf565_Supplemental_Files

## Data Availability

All data, materials, and software used in this study are available. Unprocessed FASTQ files have been deposited to the Sequence Read Archive (SRA) under the accession SRP548417. All other data are available at Figshare (https://doi.org/10.6084/m9.figshare.27281064.v1). All original code used in this study is available at Github (https://github.com/cornwero/LocalStabilityCompensation) and Zenodo (https://doi.org/10.5281/zenodo.14252189) and full datasets are available at Figshare (https://figshare.com/ndownloader/files/51111164).

## Notes

Present address: DocuSign Inc., San Francisco, CA 94105, United States

## References

[1] Fabian MR, Sonenberg N, Filipowicz W Regulation of mRNA translation and stability by microRNAs. Annu Rev Biochem. 2010; 79:351–79. 10.1146/annurev-biochem-060308-103103. 20533884

[2] Mortimer SA, Kidwell MA, Doudna JA Insights into RNA structure and function from genome-wide studies. Nat Rev Genet. 2014; 15:469–79. 10.1038/nrg3681. 24821474

[3] Statello L, Guo C-J, Chen L-L et al. Gene regulation by long non-coding RNAs and its biological functions. Nat Rev Mol Cell Biol. 2021; 22:96–118. 10.1038/s41580-020-00315-9. 33353982 PMC7754182

[4] Delebecque CJ, Lindner AB, Silver PA et al. Organization of intracellular reactions with rationally designed RNA assemblies. Science. 2011; 333:470–4. 10.1126/science.1206938. 21700839

[5] Sachdeva G, Garg A, Godding D et al. *In vivo* co-localization of enzymes on RNA scaffolds increases metabolic production in a geometrically dependent manner. Nucleic Acids Res. 2014; 42:9493–503. 10.1093/nar/gku617. 25034694 PMC4132732

[6] Siu K-H, Chen RP, Sun Q et al. Synthetic scaffolds for pathway enhancement. Curr Opin Biotechnol. 2015; 36:98–106. 10.1016/j.copbio.2015.08.009. 26322735

[7] Kumar D, An C-I, Yokobayashi Y Conditional RNA interference mediated by allosteric ribozyme. J Am Chem Soc. 2009; 131:13906–7. 10.1021/ja905596t. 19788322

[8] Penchovsky R Computational design of allosteric ribozymes as molecular biosensors. Biotechnol Adv. 2014; 32:1015–27. 10.1016/j.biotechadv.2014.05.005. 24877999

[9] Mustafina K, Fukunaga K, Yokobayashi Y Design of mammalian ON-riboswitches based on tandemly fused aptamer and ribozyme. ACS Synth Biol. 2020; 9:19–25. 10.1021/acssynbio.9b00371. 31820936

[10] Ferapontova EE, Olsen EM, Gothelf KV An RNA aptamer-based electrochemical biosensor for detection of theophylline in serum. J Am Chem Soc. 2008; 130:4256–8. 10.1021/ja711326b. 18324816

[11] Autour A, Westhof E, Ryckelynck M iSpinach: a fluorogenic RNA aptamer optimized for *in vitro* applications. Nucleic Acids Res. 2016; 44:2491–500. 10.1093/nar/gkw083. 26932363 PMC4824111

[12] Tang W, Hu JH, Liu DR Aptazyme-embedded guide RNAs enable ligand-responsive genome editing and transcriptional activation. Nat Commun. 2017; 8:15939 10.1038/ncomms15939. 28656978 PMC5493748

[13] Scott WG, Finch JT, Klug A The crystal structure of an all-RNA hammerhead ribozyme: a proposed mechanism for RNA catalytic cleavage. Cell. 1995; 81:991–1002. 10.1016/S0092-8674(05)80004-2. 7541315

[14] Ago H, Adachi T, Yoshida A et al. Crystal structure of the RNA-dependent RNA polymerase of hepatitis C virus. Structure. 1999; 7:1417–26. 10.1016/S0969-2126(00)80031-3. 10574802

[15] Kazantsev AV, Krivenko AA, Harrington DJ et al. Crystal structure of a bacterial ribonuclease P RNA. Proc Natl Acad Sci USA. 2005; 102:13392–7. 10.1073/pnas.0506662102. 16157868 PMC1224664

[16] Zhang K, Li S, Kappel K et al. Cryo-EM structure of a 40 kDa SAM-IV riboswitch RNA at 3.7 Å resolution. Nat Commun. 2019; 10:5511 10.1038/s41467-019-13494-7. 31796736 PMC6890682

[17] Ma H, Jia X, Zhang K et al. Cryo-EM advances in RNA structure determination. Signal Transduct Target Ther. 2022; 7:10.1038/s41392-022-00916-0 PMC8864457 35197441

[18] Bonilla SL, Kieft JS The promise of cryo-EM to explore RNA structural dynamics. J Mol Biol. 2022; 434:167802 10.1016/j.jmb.2022.167802. 36049551 PMC10084733

[19] Tijerina P, Mohr S, Russell R DMS footprinting of structured RNAs and RNA–protein complexes. Nat Protoc. 2007; 2:2608–23. 10.1038/nprot.2007.380. 17948004 PMC2701642

[20] Weeks KM Advances in RNA structure analysis by chemical probing. Curr Opin Struct Biol. 2010; 20:295–304. 10.1016/j.sbi.2010.04.001. 20447823 PMC2916962

[21] Boerneke MA, Ehrhardt JE, Weeks KM Physical and functional analysis of viral RNA genomes by SHAPE. Annu Rev Virol. 2019; 6:93–117. 10.1146/annurev-virology-092917-043315. 31337286 PMC6768749

[22] Juzumiene D, Shapkina T, Kirillov S et al. Short-range RNA–RNA crosslinking methods to determine rRNA structure and interactions. Methods. 2001; 25:333–43. 10.1006/meth.2001.1245. 11860287

[23] Wang X-W, Liu C-X, Chen L-L et al. RNA structure probing uncovers RNA structure-dependent biological f unctions. Nat Chem Biol. 2021; 17:755–66. 10.1038/s41589-021-00805-7. 34172967

[24] Van Damme R, Li K, Zhang M et al. Chemical reversible crosslinking enables measurement of RNA 3D distances and alternative conformations in cells. Nat Commun. 2022; 13:911 10.1038/s41467-022-28602-3. 35177610 PMC8854666

[25] Churkin A, Barash D RNA Design: Methods and Protocols. 2025; New York Springer.

[26] Zuker M Mfold web server for nucleic acid folding and hybridization prediction. Nucleic Acids Res. 2003; 31:3406–15. 10.1093/nar/gkg595. 12824337 PMC169194

[27] Reuter JS, Mathews DH RNAstructure: software for RNA secondary structure prediction and analysis. BMC Bioinformatics. 2010; 11:129 10.1186/1471-2105-11-129. 20230624 PMC2984261

[28] Lorenz R, Bernhart SH, Höner zu Siederdissen C et al. ViennaRNA package 2.0. Algorithms Mol Biol. 2011; 6:26 10.1186/1748-7188-6-26. 22115189 PMC3319429

[29] Huang L, Zhang H, Deng D et al. LinearFold: linear-time approximate RNA folding by 5′-to-3′ dynamic programming and beam search. Bioinformatics. 2019; 35:i295–304. 10.1093/bioinformatics/btz375. 31510672 PMC6681470

[30] Do CB, Woods DA, Batzoglou S CONTRAfold: RNA secondary structure prediction without physics-based models. Bioinformatics. 2006; 22:e90–8. 10.1093/bioinformatics/btl246. 16873527

[31] Sato K, Akiyama M, Sakakibara Y RNA secondary structure prediction using deep learning with thermodynamic integration. Nat Commun. 2021; 12:941 10.1038/s41467-021-21194-4. 33574226 PMC7878809

[32] Fu L, Cao Y, Wu J et al. UFold: fast and accurate RNA secondary structure prediction with deep learning. Nucleic Acids Res. 2022; 50:e14 10.1093/nar/gkab1074. 34792173 PMC8860580

[33] Sato K, Hamada M Recent trends in RNA informatics: a review of machine learning and deep learning for RNA secondary structure prediction and RNA drug discovery. Brief Bioinform. 2023; 24:bbad186 10.1093/bib/bbad186. 37232359 PMC10359090

[34] Justyna M, Antczak M, Szachniuk M Machine learning for RNA 2D structure prediction benchmarked on experimental data. Brief Bioinform. 2023; 24:bbad153 10.1093/bib/bbad153. 37096592 PMC10199776

[35] Busch A, Backofen R INFO-RNA—a fast approach to inverse RNA folding. Bioinformatics. 2006; 22:1823–31. 10.1093/bioinformatics/btl194. 16709587

[36] Lyngsø RB, Anderson JW, Sizikova E et al. Frnakenstein: multiple target inverse RNA folding. BMC Bioinformatics. 2012; 13:260 10.1186/1471-2105-13-260. 23043260 PMC3534541

[37] Ward M, Courtney E, Rivas E Fitness functions for RNA structure design. Nucleic Acids Res. 2023; 51:e40 10.1093/nar/gkad097. 36869673 PMC10123107

[38] Lee J, Kladwang W, Lee M et al. RNA design rules from a massive open laboratory. Proc Natl Acad Sci USA. 2014; 111:2122–7. 10.1073/pnas.1313039111. 24469816 PMC3926058

[39] Zhu W, Lomsadze A, Borodovsky M *Ab initio* gene identification in metagenomic sequences. Nucleic Acids Res. 2010; 38:e132 10.1093/nar/gkq275. 20403810 PMC2896542

[40] Tinoco I, Bustamante C How RNA folds. J Mol Biol. 1999; 293:271–81. 10.1006/jmbi.1999.3001. 10550208

[41] Danaee P, Rouches M, Wiley M et al. bpRNA: large-scale automated annotation and analysis of RNA secondary structure. Nucleic Acids Res. 2018; 46:5381–94. 10.1093/nar/gky285. 29746666 PMC6009582

[42] Mathews DH, Disney MD, Childs JL et al. Incorporating chemical modification constraints into a dynamic programming algorithm for prediction of RNA secondary structure. Proc Natl Acad Sci USA. 2004; 101:7287–92. 10.1073/pnas.0401799101. 15123812 PMC409911

[43] Kladwang W, Topkar VV, Liu B et al. Anomalous reverse transcription through chemical modifications in polyadenosine stretches. Biochemistry. 2020; 59:2154–70. 10.1021/acs.biochem.0c00020. 32407625 PMC8118224

[44] Citron M, Schuster H The c4 repressors of bacteriophages P1 and P7 are antisense RNAs. Cell. 1990; 62:591–8. 10.1016/0092-8674(90)90023-8. 1696181

[45] Weinberg Z, Wang JX, Bogue J et al. Comparative genomics reveals 104 candidate structured RNAs from bacteria, archaea, and their metagenomes. Genome Biol. 2010; 11:R31 10.1186/gb-2010-11-3-r31. 20230605 PMC2864571

[46] Ehrenreich IM, Purugganan MD Sequence variation of microRNAs and their binding sites in *Arabidopsis*. Plant Physiol. 2008; 146:1974–82. 10.1104/pp.108.116582. 18305205 PMC2287364

[47] Starega-Roslan J, Krol J, Koscianska E et al. Structural basis of microRNA length variety. Nucleic Acids Res. 2011; 39:257–68. 10.1093/nar/gkq727. 20739353 PMC3017592

[48] Nguyen TD, Trinh TA, Bao S et al. Secondary structure RNA elements control the cleavage activity of DICER. Nat Commun. 2022; 13:2138 10.1038/s41467-022-29822-3. 35440644 PMC9018771

[49] Song L, Axtell MJ, Fedoroff NV RNA secondary structural determinants of miRNA precursor processing in *Arabidopsis*. Curr Biol. 2010; 20:37–41. 10.1016/j.cub.2009.10.076. 20015653

[50] Rosatti S, Rojas AML, Moro B et al. Principles of miRNA/miRNA* function in plant MIRNA processing. Nucleic Acids Res. 2024; 52:8356–69. 10.1093/nar/gkae458. 38850162 PMC11317159

[51] Wostenberg C, Ceres P, Polaski JT et al. A highly coupled network of tertiary interactions in the SAM-I riboswitch and their role in regulatory tuning. J Mol Biol. 2015; 427:3473–90. 10.1016/j.jmb.2015.07.027. 26343759 PMC4624507

[52] Lasher B, Hendrix DA bpRNA-align: improved RNA secondary structure global alignment for comparing and clustering RNA structures. RNA. 2023; 29:584–95. 10.1261/rna.079211.122. 36759128 PMC10159002

[53] Lan TCT, Allan MF, Malsick LE et al. Secondary structural ensembles of the SARS-CoV-2 RNA genome in infected cells. Nat Commun. 2022; 13:1128 10.1038/s41467-022-28603-2. 35236847 PMC8891300

[54] Dang TL, Le CT, Le MN et al. Select amino acids in DGCR8 are essential for the UGU–pri-miRNA interaction and processing. Commun Biol. 2020; 3:344 10.1038/s42003-020-1071-5. 32620823 PMC7334207

[55] Nguyen TA, Park J, Dang TL et al. Microprocessor depends on hemin to recognize the apical loop of primary microRNA. Nucleic Acids Res. 2018; 46:5726–36. 10.1093/nar/gky248. 29750274 PMC6009577

[56] Diaz JP, Chirayil R, Chirayil S et al. Association of a peptoid ligand with the apical loop of pri-miR-21 inhibits cleavage by Drosha. RNA. 2014; 20:528–39. 10.1261/rna.042911.113. 24497550 PMC3964914

[57] Lee Y-Y, Lee H, Kim H et al. Structure of the human DICER–pre-miRNA complex in a dicing state. Nature. 2023; 615:331–8. 10.1038/s41586-023-05723-3. 36813958

[58] Ganser LR, Kelly ML, Herschlag D et al. The roles of structural dynamics in the cellular functions of RNAs. Nat Rev Mol Cell Biol. 2019; 20:474–89. 10.1038/s41580-019-0136-0. 31182864 PMC7656661

[59] Tomezsko PJ, Corbin VDA, Gupta P et al. Determination of RNA structural diversity and its role in HIV-1 RNA splicing. Nature. 2020; 582:438–42. 10.1038/s41586-020-2253-5. 32555469 PMC7310298

[60] Morandi E, Manfredonia I, Simon LM et al. Genome-scale deconvolution of RNA structure ensembles. Nat Methods. 2021; 18:249–52. 10.1038/s41592-021-01075-w. 33619392

[61] Rivas E Evolutionary conservation of RNA sequence and structure. Wiley Interdiscip Rev RNA. 2021; 12:e1649 10.1002/wrna.1649. 33754485 PMC8250186

[62] Rivas E, Eddy SR Secondary structure alone is generally not statistically significant for the detection of noncoding RNAs. Bioinformatics. 2000; 16:583–605. 10.1093/bioinformatics/16.7.583. 11038329

